# Assessing Oral Health Content in Non-dental Professional Association Websites in India: A Cross-Sectional Study

**DOI:** 10.7759/cureus.70270

**Published:** 2024-09-26

**Authors:** Anju James, Chandrashekar Janakiram, Vaishnav Vinodkumar, Anand Suresh, Vijay S Kumar

**Affiliations:** 1 Department of Public Health Dentistry, Amrita School of Dentistry, Amrita Vishwa Vidyapeetham, Kochi, IND; 2 Department of Periodontology, Amrita School of Dentistry, Amrita Vishwa Vidyapeetham, Kochi, IND; 3 Department of Periodontology, PSM College of Dental Science and Research, Thrissur, IND

**Keywords:** allied health personnel, india, interprofessional education, oral health, physicians

## Abstract

Introduction: There is a growing need for inter-professional education (IPE) to reduce the burden of oral diseases and address oral health disparities. Professional websites associated with inter-professional education can serve as a reliable source of information about oral health. Hence, the study was conducted to determine the prevalence of oral health content on non-dental health professional associations' websites in India.

Methods: Eighty-nine organizations were selected from five types of health professional associations. Six dental search terms were used on searchable websites. The keywords were dental, oral, dentistry, mouth, teeth, and fluoride. Websites were assessed for any oral health content.

Results: Only four websites (4.5%) had any oral health content, and all four were physician-related.

Conclusion: The study highlights the limited and inconsistent oral health content on non-dental professional association websites. Improving the availability, accuracy, and comprehensiveness of oral health information on these platforms is crucial to enhancing oral health literacy and promoting better oral health outcomes.

## Introduction

Oral health serves as a vital indicator of general health, well-being, and quality of life [1]. Molecular and immunology-based evidence establishes a connection between oral diseases and other systemic non-communicable diseases (NCDs) due to their shared major risk factors [2,3]. Meta-analytic estimates reveal a strong association between oral disorders and 28 NCDs, encompassing conditions such as obesity, asthma, inflammatory bowel disease, gastric Helicobacter pylori infection, cancer, diabetes mellitus, cardiovascular disease, neurodegenerative conditions, rheumatic diseases, and neurodegenerative problems [4]. Despite being preventable, oral diseases impose a significant health burden [1]. Integration of oral health into medical care has enhanced the quality of life, particularly for patients with chronic diseases [5].

According to the World Health Organisation's (WHO) Framework for Action on Inter-Professional Education and Collaborative Practice (2010), "Inter-professional education occurs when two or more professionals learn about, from, and with each other to enable effective collaboration and improve health outcomes" [6]. Inter-professional education (IPE) offers numerous benefits, including decreased patient costs, reduced length of stay, improved quality of patient care, and minimised medical errors [7].

Given the current global healthcare trends, there is an increasing need for inter-professional education and inter-professional practice to coordinate care for individuals with chronic medical conditions. As part of their accreditation criteria, many health professional education programmes have incorporated IPE, including oral health, into their curricula. This integration aims to foster the qualities and abilities necessary for effective collaborative practices [8].

The cumulative use of the internet has led people to rely on online sources for health information [9]. However, the quality and accuracy of the information found on websites are subject to debate, often resulting in misperceptions and incorrect information [9]. Non-dental health professionals, such as physicians, nurses, pharmacists, and other allied health professionals, play a crucial role as sources of health information for the public. However, their oral health knowledge may be limited, and they may not have received formal training in this area. Non-dental health professionals can significantly promote oral health and prevent oral diseases by providing accurate and reliable information to their patients.

The means by which non-dental healthcare practitioners acquire knowledge about oral health remains unclear [10]. A literature search revealed that continuing education opportunities regarding oral health for non-dental healthcare providers have proven challenging [11]. Professional websites associated with inter-professional education can serve as a reliable source of information about oral health.

In order to effectively improve the health and quality of life of each patient, healthcare systems require well-trained specialists who can collaborate inter-professionally [12]. Integrating oral care and expanding the roles of healthcare providers can help reduce the burden of oral diseases and address oral health disparities [13]. A desirable goal is to enhance the availability of accurate and user-friendly information about oral health through various channels and across professions. The websites of professional associations are more accessible and recognisable to their members compared to those of dentistry organisations.

A literature review demonstrates the presence of oral health content on non-dental professional association websites in developed countries like the USA [10]. However, literature regarding the availability of oral health content on non-dental professional association websites in India is lacking. Consequently, this study aims to evaluate the oral health-related information present on the websites of non-dental professional organisations in India.

## Materials and methods

The list of organisations was initially identified by identifying the key terms relevant to non-dental professional organisation websites. The search was conducted by utilising the search engine "Google." The websites listed in the search results were reviewed, and their contents were explored to confirm their relevance to the study. A list of appropriate organisations was finalised based on the evaluation and cross-referencing with the official sources.

Data extraction

Data were extracted from the selected websites using a standardised data extraction form that includes the organisation's name, organisation website, presence of an oral health-related webpage, authored oral health article(s), and links to oral health resources on other sites.

Dental terms such as dental, oral, dentistry, mouth, teeth, and fluoride were used to search for oral health content. These lay terms were chosen to reflect general information on oral diseases rather than specific oral conditions.

The websites were searched between October 2022 and December 2022. Two investigators pilot-tested the data extraction tool and reviewed several websites together to standardise the data collection protocol. The collected data were entered into an Excel spreadsheet (Microsoft® Corp., Redmond, WA). In the statistical analysis, frequency distributions were created based on the types of professional disciplines, and the oral health content was presented descriptively.

## Results

The study identified a broad range of non-dental professional organisations where oral health-related content could potentially be found. The majority of these were physician-related, covering a wide spectrum of medical specialities, from the Indian Medical Association to the Urological Society of India. Additionally, allied health-related organisations, nursing councils, pharmacy and public health organisations were included in the search. A list of non-dental healthcare professional websites searched for oral health content is presented in Table 1.

**Table 1:** List of non-dental health care professional websites searched for oral health content.

S. no.	Type	Name of the association
1	Physician related	Indian Medical Association
2	Physician related	Homoeopathic Medical Association of India
3	Physician related	Unani Medical Association
4	Physician related	Ayurveda Medical Association of India
5	Physician related	Indian Psychiatric Society
6	Physician related	Indian Association of Clinical Psychologists
7	Physician related	Dermatopathology Society of India
8	Physician related	Indian Dietetic Association
9	Physician related	Nutrition Society of India
10	Physician related	Association of Plastic Surgeons of India
11	Physician related	All India Cosmetologists and Beauticians Association
12	Physician related	Indian Society of Critical Care Medicine
13	Physician related	Paediatric Association of India
14	Physician related	Indian Academy of Paediatrics
15	Physician related	Indian Society of Human Genetics
16	Physician related	Indian Academy of Medical Genetics
17	Physician related	Geriatric Society of India
18	Physician related	Indian Academy of Geriatrics
19	Physician related	Indian Association for Geriatric Mental Health
20	Physician related	The Association of Physicians of India
21	Physician related	Indian Association of Clinical Medicine
22	Physician related	Society of Indian Radiographers
23	Physician related	Indian Radiological and Imaging Association
24	Physician related	Association of Microbiologists of India
25	Physician related	Indian Association of Medical Microbiologists
26	Physician related	The Federation of Obstetric and Gynecological Societies of India
27	Physician related	All India Ophthalmological Society
28	Physician related	Indian Neuro-Ophthalmology Society
29	Physician related	Paediatric Orthopaedic Society of India
30	Physician related	Indian Association of Paediatric Surgeons
31	Physician related	The Association of Surgeons of India
32	Physician related	Indian Association of Physical Medicine and Rehabilitation
33	Physician related	Indian Association of Aesthetic Plastic Surgeons
34	Physician related	Indian Rheumatology Association
35	Physician related	Paediatric Rheumatology Society
36	Physician related	Clinical Robotic Surgery Association
37	Physician related	Indian Society of Anaesthesiologists
38	Physician related	Indian Society of Blood Transfusion and Immunohematology
39	Physician related	Cardiological Society of India
40	Physician related	Indian Association of Clinical Cardiologists
41	Physician related	Indian Society of Otology
42	Physician related	The Association of Otolaryngologists of India
43	Physician related	Association of Emergency Physicians of India
44	Physician related	Indian Society of Electro cardiology
45	Physician related	Endocrine Society of India
46	Physician related	Academy of Family Physicians of India
47	Physician related	Indian Society of Gastroenterology
48	Physician related	Indian Association of Gastrointestinal Endo surgeons
49	Physician related	Association of Gynaecologic Oncologists of India
50	Physician related	Society for Heart Failure and Transplantation
51	Physician related	Indian National Association for the Study of the Liver
52	Physician related	Clinical Infectious Diseases Society
53	Physician related	National Integrated Medical Association
54	Physician related	Indian Association of Functional Medicine
55	Physician related	Indian Society of Oncology
56	Physician related	Indian Society of Clinical Oncology
57	Physician related	Association of Nuclear Medicine Physicians of India
58	Physician related	Indian Academy of Neurology
59	Physician related	Neurological Society of India
60	Physician related	Neuro Spinal Surgeons Association
61	Physician related	Indian Society of Nephrology
62	Physician related	Vascular Society of India
63	Physician related	Urological Society of India
64	Physician related	Indian Society for Assisted Reproduction
65	Physician related	Indian Fertility Society
66	Physician related	Indian Society for the Study of Reproduction & Fertility
67	Physician related	Indian Association of Respiratory Care
68	Physician related	The Indian Association for Bronchology
69	Physician related	Indian Society for Study of Lung Cancer
70	Physician related	Indian Association of Pathologists and Microbiologists
71	Physician related	Molecular Pathology Association of India
72	Physician related	Indian Orthopaedic Association
73	Allied health related	Central Council of Physiotherapy
74	Allied health related	All India Council of Physical Therapy
75	Allied health related	Optometry Council of India
76	Allied health related	Indian Speech Language and Hearing Association
77	Allied health related	All India Association of Anesthesia and operation theatre technologist
78	Allied health related	Council for Allied and Healthcare Professionals
79	Pharmacy related	Pharmacy Council of India
80	Pharmacy related	Indian Pharmaceutical Association
81	Nursing	Indian Nursing Council
82	Nursing	Indian Paramedical Council
83	Nursing	Paramedical Council of India
84	Nursing	All India Nursing and Paramedical Council
85	Nursing	Council of Paramedical science of India
86	Nursing	All India Paramedical Council
87	Public health	Public Health Foundation of India
88	Public health	Indian Public Health Association
89	Public health	All Institute of Hygiene and Public Health

The study surveyed a total of 89 websites from various non-dental professional organizations. The majority of these organisations were physician-related, comprising 80.9% (72 organisations). Allied health and nursing-related organisations each comprised 6.7% (6 organisations each), while pharmacy-related organisations accounted for 2.3% (2 organisations). Public health-related organisations constituted 3.4% (3 organisations). The distribution of websites viewed by type of discipline is presented in Table 2.

**Table 2:** Distribution of non-dental professional organizations by type of discipline.

Organization characteristic	All websites (n= 89)	
	Number	%
Physician related	72	80.9
Allied health-related	6	6.7
Nursing related	6	6.7
Pharmacy related	2	2.3
Public health-related	3	3.4
Total	89	100

The analysis revealed that oral health content was only on physician-related professional websites, and it primarily focused on treatment rather than preventing oral diseases. For example, the Indian Medical Association included discussions on phasing out dental amalgam and mercury instruments, as well as keynote addresses to dental students. The Association of Plastic Surgeons of India provided information on oral maxillofacial reconstructive surgery. The Indian Academy of Paediatrics included standard treatment guidelines for oral thrush, while the Indian Association of Respiratory Care highlighted interdisciplinary care during COVID-19, involving dental and allied healthcare professionals. Table 3 provides an overview of the oral health content found on non-dental health professional websites and the search terms used to locate the content.

**Table 3:** Contents of oral health in non-dental health professional websites.

S. No.	Association	Oral health content	Search term
1.	Indian Medical Association [14]	Minutes of the meeting phasing out of dental amalgam and Mercury BP instruments and thermometer on 25th February 2016 keynote address to dental students speech for dental students	Dental
2.	Association of Plastic Surgeons of India	Oral maxillofacial reconstructive surgery https://apsi.in/reconstructive-surgery.php?rsid=1 [15]	Oral
3.	Indian Academy of Paediatrics	Standard treatment guidelines for oral thrush https://iapindia.org/pdf/Ch-084-Oral-Thrush.pdf [16]	Oral
4.	Indian Association of Respiratory Care	Conference proceeding: The national voice of the vibrant respiratory therapy community will be heard at the 5th session of the thematic resource dissemination conference on 29th Sep 2021 at 05.00 pm IST. "Managing interdisciplinary care during COVID-19: dental and allied healthcare professionals’ perspective” don’t miss it!!	Dental

## Discussion

All medical professionals need access to oral health information since integrating dental health and overall health has emerged as a crucial strategy for enhancing patient and public health [17]. However, many non-dental organisations have little or no information about oral health, and the necessary emphasis on adding oral health information is lacking among non-dental health professionals.

The relevance of interdisciplinary training aligns with Lamster and Eaves' call for increased interprofessional cooperation, emphasising respect for all health disciplines, enhancing awareness of each profession's role, fostering more effective communication, and maximising safety, efficiency, and effectiveness [18]. Currently, there are limited opportunities for interprofessional training. Therefore, improving the oral health content on non-dental professional association websites is crucial to enhancing oral health literacy among non-dental health professionals.

A lack of oral health information in many non-dental organisations suggests that most of the organisations are in the pre-contemplation stage of the transtheoretical theory of behaviour change, unaware of oral health's importance to their patients and profession. Identifying these organisations allows us to move them to the contemplation and preparation stages by introducing oral health information and providing relevant messaging or links [10].

Several strategies can be implemented to address the limitations identified in this study: (a) providing training to non-dental professionals to enhance their knowledge through webinars, workshops, etc.; (b) creating awareness among the public about the availability of oral health information on non-dental professional association websites; (c) collaboration between dental and non-dental professionals to develop and regularly update oral health content.

For example, the Indian Society of Oncology could add relevant information on "cancer treatment and oral health." Chemotherapy and radiation therapy (RT) hinder the growth of new cells and disrupt the healthy balance of bacteria in the mouth. Oro-dental changes resulting from RT are caused by multiple factors, including direct damage to both hard and soft tissues, damage to salivary glands, difficulty in maintaining oral hygiene due to mucositis-induced pain and trismus, leading to radiation caries (RC), periodontal breakdown, and osteoradionecrosis (ORN) [19]. The lack of healthcare facilities with a dedicated multidisciplinary team, coordination issues among team members due to the absence of established oral care protocols, and the time required to address all existing and potential oral sources of infection before RT have always been sources of distress for treating oncologists and dental teams [20]. The availability of a time-bound protocol and/or a sequence of oral treatment strategies aimed at achieving satisfactory oral health pre-RT on associated websites can help reduce oral complications following cancer treatment.

The Indian Dietetic Association website could include "Good oral health and nutrition." Diet and nutrition have a mutual correlation with oral health [21]. Food and nutrition are impacted by oral tissues, while oral health impacts the nutrients consumed [22]. Sugar consumption has been linked to a higher risk of developing dental caries. Regular consumption of acidic foods and drinks should be avoided to prevent erosive tooth damage [23]. An interdisciplinary team of dentists, general practitioners, nurses, and dieticians must collaborate to ensure that patients are well-informed about the connection between good nutrition and oral health [24].

The Federation of Obstetric and Gynaecological Societies of India could add oral health information relevant to pregnant women, such as the association between periodontitis and poor pregnancy outcomes, including preterm birth and low birth weight [25]. Considering that poor dental health during pregnancy can negatively impact the health of both the mother and the unborn child, oral health should be seen as a crucial component of prenatal care [26].

Incorporating oral health information into non-dental health professional platforms is vital for promoting a more integrated approach to healthcare, ensuring that professionals across disciplines recognise the connection between oral and systemic health. By enhancing the visibility of oral health on these websites, we can improve overall health outcomes, encourage interdisciplinary collaboration, and promote a more holistic approach to patient care. Addressing this gap is a necessary step toward fostering interprofessional education and cooperation in the healthcare sector, ultimately benefiting both professionals and patients alike.

Limitations

The reviewed websites might not represent all non-dental health professional websites, as they may primarily include larger or more well-known organizations. The results might have varied if alternative search terms, such as 'hygienist' or the names of specific oral conditions, had been used.

## Conclusions

The findings of this study highlight the limited and inconsistent oral health content on non-dental professional association websites in India. Improving the availability, accuracy, and comprehensiveness of oral health information on these platforms is crucial to enhancing oral health literacy and promoting better oral health outcomes among the population. Collaborations between dental professionals and non-dental professional associations and regular monitoring and updating of the content can contribute to achieving this goal. Future research can explore the impact of these improvements on the general public's oral health knowledge, attitudes, and behaviours.
